# SARS-CoV-2 vaccines strategies: a comprehensive review of phase 3 candidates

**DOI:** 10.1038/s41541-021-00292-w

**Published:** 2021-02-22

**Authors:** Nikolaos C. Kyriakidis, Andrés López-Cortés, Eduardo Vásconez González, Alejandra Barreto Grimaldos, Esteban Ortiz Prado

**Affiliations:** 1 One Health Research Group, Universidad de Las Américas (UDLA), Quito, Ecuador; 2 Centro de Investigacion Genetica y Genomica, Facultad de Ciencias de la Salud Eugenio Espejo, Universidad UTE, Quito, Ecuador; 3 Latin American Network for the Implementation and Validation of Clinical Pharmacogenomics Guidelines (RELIVAF-CYTED), Madrid, Spain

**Keywords:** Infection, RNA vaccines, Viral infection

## Abstract

The new SARS-CoV-2 virus is an RNA virus that belongs to the Coronaviridae family and causes COVID-19 disease. The newly sequenced virus appears to originate in China and rapidly spread throughout the world, becoming a pandemic that, until January 5th, 2021, has caused more than 1,866,000 deaths. Hence, laboratories worldwide are developing an effective vaccine against this disease, which will be essential to reduce morbidity and mortality. Currently, there more than 64 vaccine candidates, most of them aiming to induce neutralizing antibodies against the spike protein (S). These antibodies will prevent uptake through the human ACE-2 receptor, thereby limiting viral entrance. Different vaccine platforms are being used for vaccine development, each one presenting several advantages and disadvantages. Thus far, thirteen vaccine candidates are being tested in Phase 3 clinical trials; therefore, it is closer to receiving approval or authorization for large-scale immunizations.

## Introduction

COVID-19 is caused by a new positive-strand RNA coronavirus (SARS-CoV-2), which belongs to the *Coronaviridae* family, along with the severe acute respiratory syndrome (SARS) and the Middle East respiratory syndrome (MERS) coronavirus^1,2^. Their genome encodes several non-structural and structural proteins, including spike (S), envelope (E), membrane (M), and nucleocapsid (N) proteins.^3^ The majority of the candidate vaccines for COVID-19 that employ administration of viral antigens or viral gene sequences aim to induce neutralizing antibodies against the viral spike protein (S), preventing uptake through the human ACE2 receptor and, therefore, blocking infection^4^. However, a growing body of literature highlighting the importance of cellular responses on the recovery of COVID-19 patients^5–7^ has promoted not only the use of vaccine strategies that favor the induction of T cell mediated responses, but also the screening of their production in clinical trial participants. On the other hand, the strategies using whole virus -either attenuated or inactivated- aspire to induce a broader, more heterologous polyclonal response against several viral antigens.

Since the publication of the genome sequence of SARS-CoV-2, on January 11th, 2020, an endeavor of unprecedented speed and magnitude set out to develop a vaccine against the disease. Early scientific opinions predicted that it would take at least a year to a year and a half to get a SARS-CoV-2 vaccine approved for use in the United States. Still, recent advances on the field have made possible the issuing of emergency use authorizations (EUAs) by several national and international drug regulation agencies for different vaccine candidates against SARS-CoV-2 in less than a year since the virus genome sequence was released. An ideal SARS-CoV-2 vaccine should meet the following requirements: protect not only from severe disease but also thwart infection in all vaccinated populations, including less immunocompromised individuals, elicit long term memory immune responses after a minimal number of immunizations or booster doses, the manufacturing company should be able to ramp up production to produce billions of doses annually and have the potential to make it easily accessible for worldwide vaccination campaigns at an affordable cost and at limited time^8^.

Four different initiatives are among the essential sources of funding that enabled the development of several SARS-CoV-2 vaccine candidates. One early funding source was the Coalition for Epidemic Preparedness Innovations (CEPI), a non-profit global partnership aiming to provide funding for vaccines to stop emerging epidemics. Another vital injection of funding came from the Biomedical Advanced Research and Development Authority (BARDA), which has allocated several millions of dollars from BARDA to leading vaccine candidates and other COVID-19 promising treatments. The European Union Vaccine program has a joint effort underway to purchase vaccines for the EU countries. This entity has already signed contracts with six vaccine developers, including Pfizer and BioNTech, Sanofi-GSK, Curevac, AstraZeneca and the University of Oxford, Johnson & Johnson and Moderna. More recently, the US government’s Operation Warp Speed invested more than a billion dollars to finance the development of 8 leading vaccine candidates to accelerate their evaluation, approval, and manufacture for the US. Finally, Gavi, a global access vaccine alliance, CEPI, and the World Health Organization (WHO) have launched the COVAX (Coronavirus Vaccine Access) initiative to ensure equitable access of SARS-CoV-2 vaccines to non-self-financed countries that lack the resources to get early access to these vaccines otherwise.

According to WHO on January 5th, 2021, there are 63 candidate vaccines in human clinical trials and more than 172 candidates in preclinical development worldwide^9^. Among the 60 clinically evaluated vaccines we find 13 leading candidates that are already carrying out or entering Phase 3 clinical trials^10^ in an unprecedentedly expeditious vaccine development effort.

**Fig. 1:**
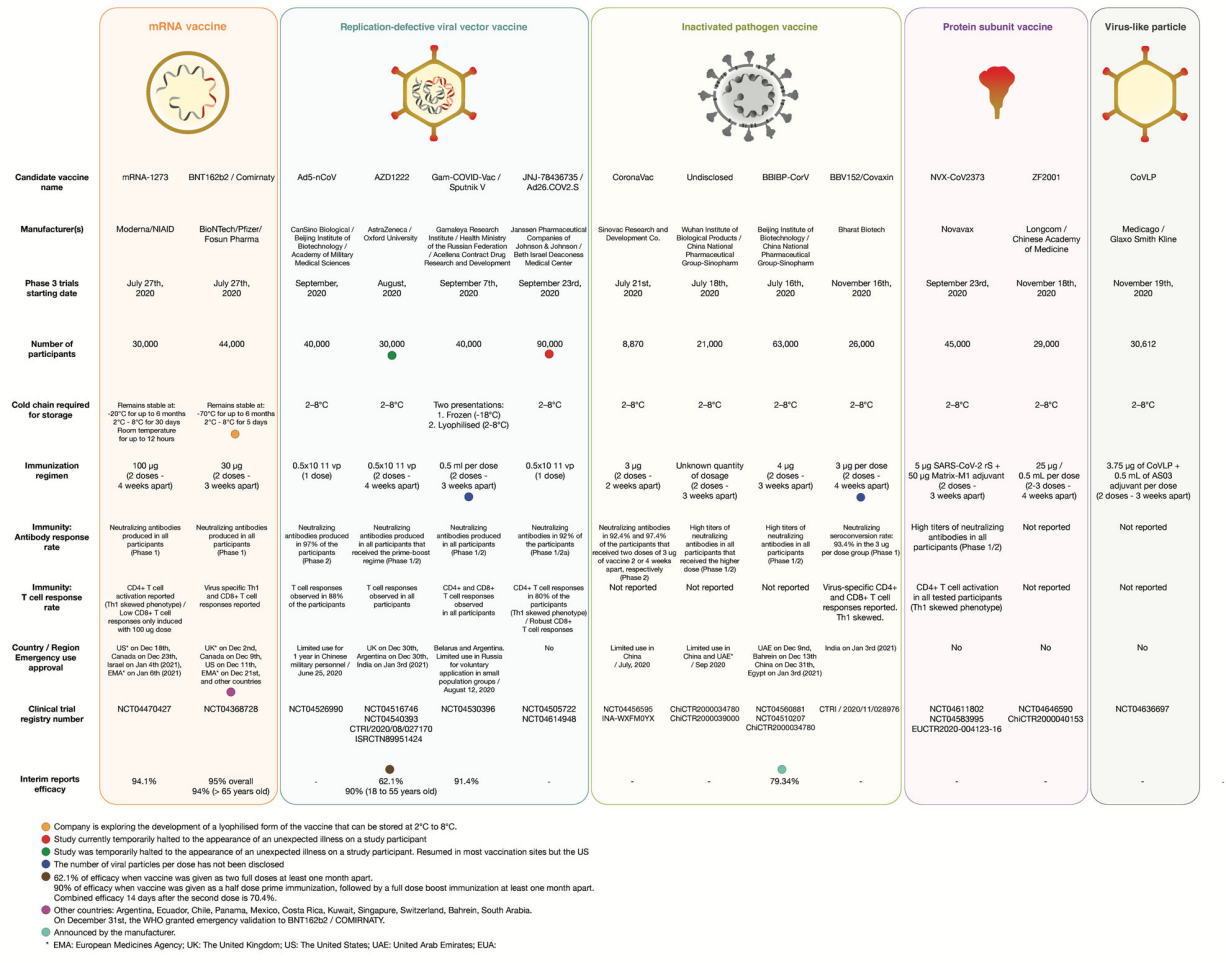
Most advanced SARS-CoV-2 vaccine candidates. The vaccine candidates are grouped according to the platform technology used for their development: mRNA vaccines, replication-defective viral vector vaccines, inactivated pathogen vaccines, protein subunit vaccines, and virus-like vaccines. Manufacturer name, Phase 3 trial data, immunogenicity and current status information are herein detailed. EMA European Medicines Agency, UK The United Kingdom, US The United States, UEA United Arab Emirates.

Platform technologies have been employed by different research groups to develop their vaccine candidates. However, it comes as no surprise that the first candidates to enter Phase 3 clinical trials are using fast deployment strategies, namely nucleic acid platforms, non-replicating viral vectored platforms, inactivated virus or recombinant subunit vaccines (see Fig. 1). Other traditional vaccine development strategies, such as attenuated virus vaccines, although historically leading to very successful vaccines against viral diseases^11,12^ require long cell culturing processes to achieve attenuated strains. It is quite possible that the second generation SARS-CoV-2 vaccines will demonstrate the capacity to elicit more robust and longer memory responses with just one immunization^13^. Herein, we discuss the different strategies that were used for vaccine development and we provide an overview of the current leading vaccine candidates against SARS-CoV-2.

## Vaccine development strategies and platforms

Since May 14th of 1796, when Edward Jenner performed the emblematic experimental inoculation of an 8-year-old boy with pus obtained from a milkmaid infected with cowpox that resulted in his immunization against smallpox, vaccination has been proven to be a successful story in Medicine. Traditional vaccine development strategies, though proven to be efficient for a number of pathogens, are slowly giving space to more sophisticated techniques involving recombinant DNA technology, adding new options in vaccine design strategies^14^. Each of these strategies presents their own set of advantages and challenges, as shown in Table 1. However, there are two main goals that any vaccine strategy needs to achieve: the safety of the vaccine and the production of robust adaptive immune responses that lead to long term protection against several strains of the pathogen with -ideally- one dose of the vaccine.

**Table 1:** Overview of the advantages and disadvantages of the major vaccine strategies and examples of licensed vaccines developed with these methods.

	Attenuated live pathogen vaccines	Inactivated pathogen vaccines	Protein Subunit vaccines	Polysaccharide vaccines	Conjugate vaccines	Virus-like particles vaccines	Viral-vectored vaccines	Nucleic acid vaccines
Advantages	Usually produce robust cellular and humoral immune responses with only one dose. Long-lasting immune responses (sometimes lifelong).	Safety, as the pathogen is dead. Transport and storage.	Safety during production. Can be safely administered to immunosuppressed people. No infectious agent handling is required.	Provides an alternative for vaccines against pathogens with an abundance of polysaccharide antigens (mostly bacteria).	Enhances the poor immunologic responses produced by polysaccharide vaccines as it induces T-dependent responses.	They combine the efficacy of attenuated vaccines and the safety of subunit vaccines. Scalability of production. Their size makes them ideal for uptake by APCs^a^.	Can induce robust humoral and cellular responses with a single dose. Good safety profile.	Scalability. Fast design and development. Extremely safe. No infectious agent handling is required. Can induce humoral and cellular responses.
Disadvantages	Safety issues in immunosuppressed people. Difficulty in achieving weakened strains. Development time. Needs refrigeration.	Large quantities of the pathogen need to be processed. The inactivation process can affect the antigen immunogenicity. Antibody titers reduce over time. Need several booster doses. Do not produce cellular responses.	Small size of antigens diminishes their uptake by APCs^a^. Low immunogenicity. Need several booster doses and adjuvants. Do not elicit cellular responses. Antigen integrity needs to be confirmed. Production limited by antigen production scalability.	Boost doses seldomly enhance the responses. Only IgM isotype and IgG2 subtype are induced leading to limited antibody mediated effector functions. Poor memory responses. Works poorly on children.	Absence of cellular responses. Adjuvant and booster doses needed.	The assembly of the particles is sometimes challenging.	Pre-existing immunity against a human viral vector can attenuate immune responses. Some candidates require storage at < −20 °C.	Currently, there is no nucleic acid vaccine approved. DNA vaccines require a special delivery platform. mRNA vaccines exhibit instability and require storage at < −20^o^C.
**L**icensed vaccines that use this strategy^d^	Oral Polio, Yellow fever, Chickenpox, Mumps, Measles, Rotavirus, Rubella, Vaccinia, BCG^c^	Rabies, Polio, Hep A	Hep B, Hep C, Influenza, Acellular pertussis vaccine, HPV^b^	Pneumococcal polysaccharide vaccine (PPSV or PPV-23), *Neisseria meningitidis* polysaccharide vaccine (meningococcal vaccine)	*Streptococcus pneumoniae* vaccine, *Neisseria meningitidis* conjugated vaccine (meningococcal vaccine), Typhoid vaccine, *Haemophilus influenzae* type b vaccine	HPV^b^, Hep B	Ebola	-

^a^APCs: antigen-presenting cells.

^b^HPV: human papilloma virus.

^c^BCG: Bacillus Calmette-Guerin.

^d^SARS-CoV-2 vaccines not included.

The vast majority of approved vaccines was traditionally focused on the induction of strong protective neutralizing antibodies against the target pathogen, thus aiming to confer sterilizing immunity in vaccinated individuals. Sterilizing immunity describes the immune status whereby virus infection of the host is totally inhibited and, therefore, disease and further transmission of the virus prevented. It differs from innate trained or T-cell mediated immunity that allows for infection, but efficiently controls and subsequently eradicates the pathogen. Sterilizing immunity is quite rare especially against viruses that infect the lower mucosa of the respiratory tract, such as the influenza virus or different coronaviruses^15^ Yet, a growing body of evidence suggests that T-cell mediated responses against SARS-CoV-2 are extremely important and more long-lasting than B-cell immunity^16,17^. Therefore, vaccine strategies that induce strong cellular responses apart from humoral immunity present a significant advantage in the present pandemic.

### Attenuated pathogen vaccines

The traditional vaccine strategy of attenuated pathogen administration was first developed on bacteria by Pasteur in 1880^18^. In the immediate post-World War II years Enders and colleagues developed prolonged virus culture techniques to attenuate viral strains an explosion of interest vaccine development took place and led to the production of some of the vaccines against measles, mumps, rubella, and poliomyelitis^19^. The success of this strategy mainly lies in the fact that in administering a live version of the pathogen it mimics almost precisely the natural infection without causing disease. An illustrative example of their efficacy is the fact that in the years preceding the production and implementation of the measles vaccine in the United States the mean incidence of measles was 900,000 cases per year in contrast to the fewer than 100 cases per year reported in recent decades^20^.

Normally, to achieve attenuated strains of a pathogen, exhaustively long cell or animal cultures are required. By replicating in a foreign host, the wild-type virus needs to accumulate mutations that adapt it to the new host and potentially impair its virulence in the human host, and this process can take years or yield poorly attenuated strains that can rapidly revert genetically to the initial wild-type genotype. In this regard, coronaviruses are known to frequently recombine in nature, further complicating the development of an attenuated live vaccine against SARS-CoV-2, as the attenuated strain could recombine with other wild coronaviruses resulting in a fully virulent strain^21^. Moreover, pre-existing cross-reactive immunity from natural contact with other human coronaviruses may potentially limit the efficacy of SARS-CoV-2 vaccines using this platform. Another drawback of this strategy is that attenuated vaccines cannot be applied to immunocompromised individuals as the attenuated agent finds the niche to multiply in an uncontrolled fashion and, in rare cases, revert to a wild type phenotype causing severe disease. Paradoxically, attenuated pathogen vaccine strategies have given us the fastest vaccine produced so far. In 1963 Maurice Hilleman’s daughter Jeryl Lynn developed parotitis. Her father, a vaccinologist working for Merck & Co., isolated the mumps virus from her throat. Over the next few months, he systematically “weakened” the isolated strain by passaging it in cell cultures. In the following 2 years human trials using the attenuated strain were conducted, and Merck licensed the vaccine in December 1967.

**Fig. 2:**
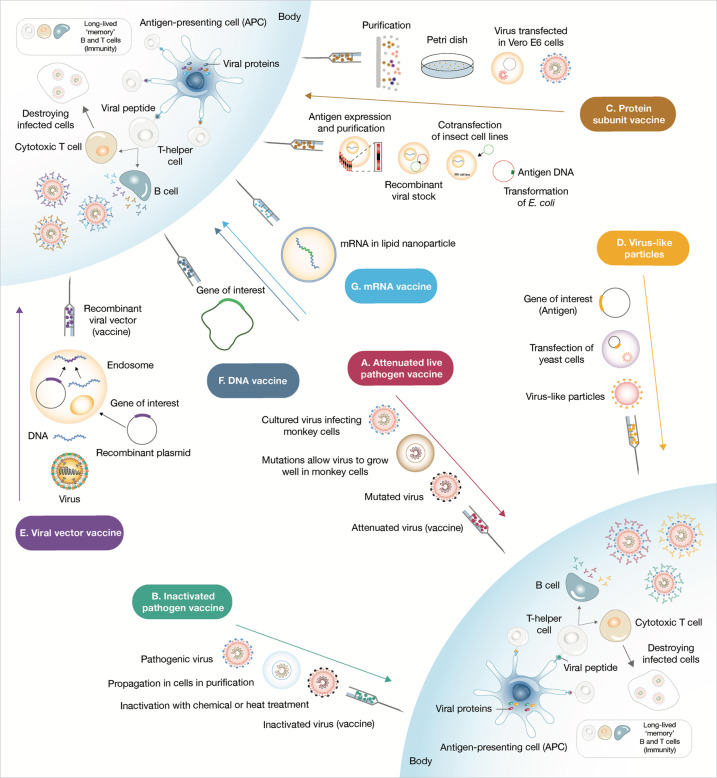
Overview of the strategies used for vaccine development and delivery. **A** Attenuated live pathogen vaccine strategies consist in administering a debilitated form of live pathogen. Lengthy cell culture passaging in non-human cell lines or animals decreases the virulence of the pathogen. This type of vaccines usually elicits robust and long-term memory immune responses after a single dose. **B** Inactivated pathogen vaccines contain whole pathogen that has been submitted to heat or chemical treatment inactivation. **C** Subunit vaccines are prepared either from antigen purification of pathogens replicated in cell cultures or from recombinantly expressed antigens. These vaccines commonly require adjuvant addition in order to deliver danger signals to antigen-presenting cells and provoke robust immune responses. **D** Virus-like particles can be self-assembled in and released from recombinant yeast cells or other expression systems such as the vaccinia virus expression system or even tobacco plants transfected with tobacco mosaic virus. **E** Viral vector vaccines use a genetically manipulated measles or adenoviral platform to express a foreign antigen commonly resulting in robust cellular and humoral response. **F**, **G** Lastly, nucleic acid (DNA and mRNA) vaccines are very quick to produce, yet were untested as successful human vaccine strategies. The nucleic acid codifying for an immunogenic protein of the pathogen once administered is captured by antigen-presenting cells that use it to express and present the antigen. These vaccines are predicted to have minor safety issues as nucleic acid is swiftly degraded within the human body.

Not surprisingly, no SARS-CoV-2 vaccine candidate using this strategy has initiated clinical trials. The attenuated pathogen vaccine strategy is detailed in Fig. 2A.

### Inactivated pathogen vaccines

A few years after the attenuated cholera vaccine produced by Pasteur, Salmon and Smith were introducing the method of heat, gamma radiation or chemical treatments (i.e., formalin, β-propiolactone) to inactivate pathogen vaccines aiming to tackle these rare events of severe adverse effects after live-attenuated pathogen administration^22,23^. Inactivated pathogen vaccines use a dead form of the pathogen, thus ensuring a better safety profile than live attenuated vaccines. However, chemically, irradiated or heat-inactivated pathogens, sometimes lose their immunogenicity rendering this strategy less efficacious than live attenuated pathogen immunization. Accordingly, inactivated pathogen vaccines often fail to induce cellular adaptive responses unless and thus require the addition of adjuvants, specific compounds that act as stimulants of immune cells and amplifiers of immune responses, is required. The inactivated pathogen vaccine strategy is detailed in Fig. 2B.

### Subunit vaccines

The principle underlying the development of subunit vaccines was based upon the observation that do not need to administer the entire pathogen to elicit strong immune responses, but merely an immunogenic fragment. Protein subunit vaccines, polysaccharide and conjugated vaccines and virus-like particle vaccines are all considered to be different forms of subunit administration strategies that differ in the chemical nature of the antigen administered, the platform used to administer the antigen and the necessity to use an adjuvant to potently activate the immune system.

#### Protein subunit vaccines

The first forms of developed subunit vaccines aimed to harness early on the ability of protein antigens to elicit germinal centre reactions and lead to high affinity, isotype-switched immunoglobulins.

Protein subunit vaccines are generated through recombinant synthesis of protein antigens or protein isolation and purification methods after cultivating large amounts of the pathogen. This strategy eliminates the possibility of severe adverse effects, but frequently raises the necessity to increase booster doses and optimize the adjuvant added to achieve stronger and more durable immunization. The administered antigen is uptaken by adjuvant activated antigen-presenting cells (APCs) and presented to adaptive immune cells.

One of the earliest examples of an acellular vaccine was the anthrax protective antigen developed in the early 1960s but perhaps the most famous response of this strategy are the subunit vaccines for influenza^24–26^.

The explosion of genetic engineering observed in the last two decades of the 20th century resulted in the capacity to clone and ramp up antigen production in vitro. Such techniques permitted the production of large quantities of the hepatitis B surface antigen in yeast cells, a breakthrough that led to the production of the Hepatitis B vaccine^27^.

A plethora of protein subunit candidates against SARS-CoV-2 is currently in human clinical trials. Each one of these candidates is using different immunogens, principally different forms of the entire Spike protein or its receptor binding domain (RBD), the region of the S protein that mediates viral binding to the ACE2 receptor of target host cells. Upon binding to the host cell ACE2 receptor the prefusion conformation of the S protein undergoes an extended conformational change to a highly stable post fusion conformation that permits the fusion between the viral particle and host cell membranes^28^. As a general rule, prefusion-stabilized viral glycoproteins are usually more immunogenic, thus being more attractive vaccine targets^29–31^. The protein subunit vaccine strategy is detailed in Fig. 2C.

#### Virus-like particle vaccines

‘Virus-like particle’ (VLP) vaccines explore the immunogenicity and safety of empty virus particles presenting several copies of the same antigen on their surface. These are designed to mimic the virus structure, thereby triggering strong immune responses against the antigen(s) presented on their surface; they have good safety profiles because they lack the pathogen’s genetic material. This characteristic, however, represents a complexity in their development because their assembly can be technically challenging. In the mid-1990s, the work of two independent groups led to the self-assembly of L1 human papilloma virus (HPV) protein into VLPs provided the platform for the GlaxoSmithKline and MERCK vaccine design for HPV^32,33^.

One virus-like particle candidate for SARS-CoV-2 developed by Canada based Medicago Inc. is already in Phase 3 clinical trials. This candidate displays the stabilized prefusion form of the SARS-CoV-2 S protein on the surface of self-assembling VLPs. The virus-like particle vaccine strategy is detailed in Fig. 2D.

### Viral-vectored vaccines

Viral vectors represent one of the latest strategies for vaccine development. Different viruses are modified to reduce their virulence and—usually—their replication potential but maintain their capacity to infect human cells. These are designed to deliver the pathogen’s genetic information to immune cells in order to express and present antigenic proteins to lymphocytes. Adenovirus, measles, and vesicular stomatitis virus (VSV) vectors are commonly used for such designs which have been shown to provoke robust immune responses with a single administration.

So far, there are two members in this group that has received approval. On one hand, a recombinant vesicular stomatitis virus (rVSV) vector that contains the genetic information codifying for a glycoprotein of Ebola. This vaccine named “rVSV-ZEBOV” or Ervebo^34^ received approval in December 2019. On the other hand, an heterologous adenovirus 26 (Ad26) and Modified Vaccinia Ankara (MVA) vectored vaccine, also against the Zaire strain of Ebola virus, commercially called Zabdeno and Mvabea (‘Ad26.ZEBOV + MVA-BN-Filo’), was granted marketing authorization by the European Medicines Agency (EMA) for use in the European Commission on July 1, 2020^35^.

An observed complication in the employment of viral vectored vaccines is pre-existing immunity against the vector that can impair the magnitude of the elicited immune responses^36^. In line with this, in a multiple vaccination regimen (e.g., prime-boost vaccinations) antibodies against the viral vector produced after the prime vaccination can decrease the immunogenicity of booster administrations^37^. Using less common vector serotypes or non-human viral vectors (eg. adenovirus derived from chimpanzees) can help circumvent this immunological conundrum.

There are two broad viral vector groups used for vaccine production, namely replication-competent and replication-defective viral vectored vaccines. Replication-competent vectors need lower dose to elicit strong responses as the multiplying vector can result in enhanced antigen presentation. Conversely, replication-defective vectors should be administered in higher dosages since they are devoid of a self-propagation capacity. However, this last characteristic allows translating into safer platforms.

Several replication-competent and replication-defective SARS-CoV-2 candidate vaccines are in clinical trials^38^. Nonetheless, only replication-defective counterparts are currently being tested in Phase III clinical trials. The viral-vector vaccine strategy is detailed in Fig. 2E.

### Nucleid acid vaccines

One of the recent trends in vaccine development is the development of nucleic acid platforms that encode for pathogen antigens. There are no approved DNA or mRNA vaccines for use in humans yet. However, there are DNA-based licenced by the USDA for veterinary use. Among them, there is a vaccine against West Nile Virus in horses^39^ and one against canine melanoma^40^. Nucleic acid vaccines are potent inducers of both humoral and cellular adaptive immune responses and are very fast to deploy since the only ingredient required for their production is the genetic sequence that encodes for a viral antigen and a delivery platform. Their fast-track design and production allowed them to emerge as spearhead candidates against the new coronavirus SARS-CoV-2 (Fig. 1). Since the DNA and mRNA molecules have different stability and, also include different steps that lead to antigen production these two platforms present different challenges that are analyzed in the following sections.

#### DNA vaccines

DNA vaccines can have different routes of application. They can be delivered intradermally whereby a short electric pulse (electroporation) optimizes their uptake by cutaneous antigen-presenting cells (APCs) such as macrophages, monocytes, and dendritic cells that will process and present them to naïve T cells in secondary lymph organs, thus raising cellular adaptive immune responses. Newly synthesized antigen will also arrive in these organs and initiate naïve B cell activation that will result in antibody production. Subcutaneous administration of DNA will lead to fibroblast and keratinocyte uptake. These cells will subsequently synthesize and release the antigen that can be recognized and phagocytosed by APCs. Transdermal administration of DNA will primarily engage by tissue-resident Langerhans cells that will express, process, and present the transgene. On the other hand, DNA administered via intravenous injection will systematically reach secondary lymphatic organs, whereas intramuscular application of a DNA vaccine enhanced by electroporation can principally lead to myocyte delivery. Myocytes will subsequently synthesize and secrete the nascent antigen that will subsequently be uptaken by APCs that can initiate adaptive immune responses. Finally, nebulisations of DNA vaccines will result in activation of pulmonary APCs inducing mucosal immunity, whereas, in a similar fashion, orally administered DNA in the form of bacterial plasmids will provoke uptake by intestinal epithelial cells. These mediators will express large amounts of the antigen leading to its uptake by intestinal APCs and subsequent presentation in gut associated lymphoid tissues (GALTs), such as Peyer’s patches^41^.

DNA molecules are generally quite stable, permitting the storage of DNA vaccines at +4 °C, thereby simplifying the distribution of this type of vaccines (Fig. 2F).

#### mRNA vaccines

The delivery of mRNA vaccines follows the same concept as DNA vaccines with the difference that mRNA only needs to reach cytoplasmic or endoplasmic reticulum ribosomes in order to be translated into protein. mRNA molecules can therefore be administered encapsulated in lipid nanoparticle (LNP) vectors that can encapsulate efficiently nucleic acid and potently enable tissue penetration to facilitate genetic information delivery in host cells so that foreign antigen protein synthesis can initiate. The subsequent induction of immune responses is similar to the induction of DNA vaccines.

However, the mRNA molecules are significantly more unstable than DNA. Hence, mRNA vaccines commonly require temperatures between −70 °C and −20 °C for long-term storage that complicate the distribution logistics of these kind of vaccines. Addition of specific mutations and stabilizing chemical modifications to mRNA vaccine molecules are specifically aiming to tackle these problems^42^ (Fig. 2G); allowing for short-term storage (up to 6 months) of mRNA vaccine candidates at temperatures between 2 and 8 °C (more details on storage temperatures of leading SARS-CoV-2 vaccine candidates are presented in Fig. 1).

## Current vaccine candidates against SARS-CoV-2 in Phase 3 clinical evaluation

### Nucleic acid vaccines

#### mRNA vaccines

##### mRNA-1273 (Moderna/US NIAID)

Boston based Moderna Therapeutics partnered up with the National Institute of Allergy and Infectious Diseases (NIAID) to produce the first vaccine candidate that entered clinical trials in 63 days after the genome sequencing of SARS-CoV-2. The vaccine is based on an mRNA molecule that contains the information for the synthesis of the stabilized pre-fusion form of the SARS-CoV-2 Spike (S) protein encapsulated in a lipid nanoparticle (LNP) vector that enhances uptake by host immune cells. The administered mRNA uses the host cell transcription and translation machinery to produce the viral antigen that is afterward presented in T lymphocytes and is also directly recognized by B lymphocytes of the host, thereby initiating an adaptive immune response directed against the S protein of the virus.

In preclinical studies, administration of mRNA-1273 induced potent humoral and cellular responses in BALB/cJ, C57BL/6J, and B6C3F1/J mice that received two intramuscular doses of 1 μg mRNA-1273, 3-weeks apart. Apart from induction of high levels of virus-specific antibodies, administration of mRNA-1273 was found to elicit neutralizing antibodies against as assessed by a pseudovirus-neutralizing test. Immunized mice also developed robust Th1-skewed CD4+ and CD8+ antigen-specific responses. Additionally, a mouse adapted version of SARS-CoV-2 containing two mutations that allow for binding to mouse angiotensin-converting enzyme 2 failed to infect 6 out of 7 vaccinated BALB/cJ mice in their upper and lower respiratory tract (33).

Dosing of the first volunteers with mRNA-1273 began on March 16th in 45 healthy volunteers ranging from 18 to 55 years old that received three different doses, namely 25 μg, 100 μg and 250 μg of RNA in a prime-boost fashion. The second vaccination was administered 28 days after the first one (Fig. 1).

The Phase 1 trial report described a dose-dependent humoral response and the production of neutralization antibodies in titers similar to the ones detected in COVID-19 convalescent sera^43^. Moreover, the two lower doses elicited strong CD4+ T cell response with a minimum expression of T helper 2 (Th2) cytokines that were found to be detrimental during SARS and MERS vaccine development efforts^44,45^. On the other hand, CD8+ T cell responses were only elicited by the 100 μg -medium level- dose. The mRNA-1273 was generally well-tolerated, and no stage 4 (incapacitating or potentially lethal) adverse effects were reported. Adverse events, comprised mostly myalgia, fatigue, headache, chills, and pain around the injection site, were more frequent after the second immunization and were more prominent in the high dose group (250 μg).

Additional results from a small Phase 1 study in 40 older adults, which were divided in two age groups (56–70 years or ≥71 years) were recently published^46^. Participants were administered two doses of either 25 μg or 100 μg of mRNA-1273 that have previously shown to exhibit a higher tolerance profile. The immunogenic profile of both age groups was quite similar to that reported in the group 18–55, suggesting that this vaccination strategy might be equally immunogenic in more vulnerable and usually less immunocompetent age groups. The 100 μg dose was found to be more immunogenic supporting its use in a Phase 3 vaccine trial.

After completing a Phase 2 vaccination trial in 300 young and 300 older adults administrated either the 25 or the 100 µg doses, mRNA-1273 entered a Phase 3 efficacy trial on July 27th (Clinical Trial Identifier: NCT04470427). This trial involves the enrollment of 30,000 participants in the U.S. half of whom will receive 2 doses of 100 µg of mRNA-1273 and the other half a placebo in a randomized double-blind placebo-controlled study. The primary endpoint of the study is the prevention of symptomatic COVID-19 with secondary endpoints including prevention of infection by SARS-CoV-2 and prevention of hospitalization from COVID-19.

On November 18th, Moderna announced that their vaccine candidate met its primary efficacy endpoint after reviewing the first interim analysis of their Phase 3 clinical study^47^. This initial analysis, set 14 days after the second vaccination, includes 95 confirmed COVID-19 cases among the participants, 90 of whom belonged in the group that received the placebo and 5 in the group that received mRNA-1273. The case split calculations reveal an initial 94.5% efficacy of the vaccine candidate. Another important observation was that of 11 severe cases analyzed none had occurred in the mRNA-1273 vaccinated group, suggesting that possibly mRNA-1273 protects from severe COVID-19, although more data are needed to establish certainty. As to the safety profile of mRNA-1273 mostly mild to moderate events were reported. The most frequent severe adverse effects were soreness at the injection site after the first dose (2.7%), and fatigue (9.7%), myalgia (8.9%), arthralgia (5.2%), headache (4.5%), pain (4.1%), and redness at the injection site (2.0%) after the second dose. Overall, these effects were described as short-lived.

On a second press release again on November 16th, Moderna revealed that after additional testing, their vaccine candidate was found to remain stable at 2° to 8 °C for 30 days, at −20 °C for up to 6 months and at room temperature for up to 12 h, thus tackling one of the major challenges presented with mRNA vaccines that is the logistics of its distribution in rural areas and the need of specialized refrigerators^48^.

On November 30th, Moderna announced the results from the primary efficacy analysis of their Phase 3 study involving 196 cases of confirmed COVID-19^49^. Among the 30 individuals that developed severe disease, none had been immunized with the vaccine candidate, suggesting that mRNA-1273 strongly protects against severe disease. Moreover, out of the 196 COVID-19 confirmed cases only 11 belonged in the mRNA-1273 arm of the study, yielding a point estimate of vaccine efficacy of 94.1% 2 weeks after the second dose. Efficacy was reported to be consistent across age, race, and ethnicity, whilst no differences were reported on the previously released safety profile of the candidate. The company has therefore reached both endpoints of 151 confirmed cases and an average two-month follow-up of the participants set in the design of the study and on December 18th, the US FDA has granted a EUA of the vaccine in individuals >18 years of age, followed by Health Canada on December 23^rd^ ^50,51^. On December 30th, the safety and efficacy results from the Phase 3 trial of mRNA-1273 were published in the New England Journal of Medicine, confirming the vaccine candidate’s 94.1% efficacy and safety profile^46^. On January 4th, 2021, Israel also approved Moderna’s vaccine candidate, and later on, the European Medicines Agency (EMA) recommended the vaccine for authorization^52^.

##### mRNA-BNT162b2/Comirnaty (Pfizer/BioNTech/Fosun Pharma)

The second candidate mRNA platform is BNT162b2 developed by Pfizer in collaboration with German based BioNTech (an abbreviation for Biopharmaceutical New Technologies) and Shangai-based Fosun Pharma. BioNTech initially developed and tested four modified mRNA-based (modRNA) vaccine candidates designed to be administered in two vaccinations 3-weeks apart and, upon insertion to host cell cytoplasm, instructs immune cells to make several copies of the full-length SARS-CoV-2 spike protein.

Preliminary data in non-human primate models revealed that immunization of BALB/c mice with candidate BNT162b2 induced strong humoral and cellular anti-SARS-CoV-2 responses characterized by high titers of specific neutralizing antibodies and activation of CD8+ and CD4+ T lymphocytes that exhibited a Th1 skewed phenotype. Neutralizing antibody levels were assessed with a VSV-based GFP-encoding vector that had been pseudotyped to present the SARS-CoV-2 S protein on its envelope. When highly diluted sera of immunized mice were preincubated with the VSV/SARS-CoV-2 pseudovirus, they strongly inhibited Vero-76 cells infection as assessed by reduced GFP fluorescence, suggesting that immunized mice displayed high titers of SARS-CoV-2 neutralizing antibodies. Rhesus macaques immunized with two intramuscularly administered doses of BNT162b2 developed high titers of antibodies that were found to neutralize wild type SARS-CoV-2 and developed potent Th1-biased responses. When challenged with the USA-WA1/2020 strain of SARS-CoV-2 immunized animals showed no viral RNA replication in their lungs, whereas in nasal swabs detection was only found in samples obtained the day following the viral challenge and in none of the samples obtained after day 3 post-challenge. Finally, vaccinated animals did not show any clinical signs of disease^53^.

Results from Phase 1 randomized placebo-controlled clinical trials showed that BNT162b2 generates minimum side effects both in younger (18–55 years old) and older (65–85 years old) participants^53^. Also, two different candidates were evaluated in these trials, namely BNT162b1 and BNT162b2. Both candidates induced the production of similarly high dose-dependent neutralizing antibody titers against SARS-CoV-2 in the inoculated participants. Indeed, the neutralizing antibody titers were higher or equal to SARS-CoV-2 convalescent sera. Nevertheless, BNT162b2 demonstrated less systemic reactogenicity in older adults and the 30 µg dose of this candidate was, therefore, selected for large-scale Phase 2/3 efficacy studies^54^. T lymphocyte responses were not initially reported for this specific candidate, but based on a previous study on the immunogenicity of BNT162b1^55^ significant activation of specific CD8+ T cell and CD4+ Th1-skewed populations are expected. Indeed, a two-dose immunization with 30 µg/dosis of BNT162b1 was found to induce high titers of neutralizing anti-SARS-CoV-2 antibodies and virus-specific Th1 and CD8+ T cell responses^56^. Phase 2/3 safety and efficacy randomised placebo-controlled trials will be conducted in 43,488 volunteers, including individuals with underlying chronic conditions and different genetic backgrounds (Clinical Trial Identifier: NCT04368728).

The trial’s primary endpoint is prevention of COVID-19, and secondary endpoints include prevention of severe COVID-19 and prevention of infection by SARS-CoV-2. The trial is designed to be carried out in 166 clinical investigational sites around the world in at least 3 different continents. The manufacturing capacity of Pfizer will permit a global supply of up to 50 million doses by the end of 2020 and 1.3 billion doses by the end of 2021 if their vaccine candidate achieves authorization (Fig. 1).

In a press release issued on November 18^th^, Pfizer and BioNTech announced that final interim analysis data suggest that their candidate demonstrated a 95% efficacy against COVID-19 one week after administrating both immunizations. These data are based on the evaluation of 43,448 participants, 170 of whom developed COVID-19 in the evaluation window. Among them, 162 belong in the placebo group and 8 in the group that was immunized with the vaccine candidate. According to the press release vaccine efficacy was consistent across age, gender, race, and ethnicity demographics. Furthermore, observed efficacy in vaccinated participants over 65 years of age was over 94%. Concerning the safety profile of BNT162b2, the vaccine candidate was well tolerated across all populations as no serious safety concerns were reported. The more frequent Grade 3 events were fatigue presented at 3.8% and headache at 2.0% of the vaccinated participants^57^. According to these results, the company considers that the safety data milestone required by U.S. Food and Drug Administration (FDA) for EUA has been met and on November 20th, Pfizer and BioNtech became the first companies that submitted a request for an EUA of a SARS-CoV-2 vaccine to FDA^58^. On December 2nd, BNT162b2 received an EUA from Medicines and Healthcare products Regulatory Agency (MHRA), the United Kingdom’s drug regulator and vaccine administration began on December 8th in the UK, followed by an authorization granted by the Canadian medicines regulatory agency on December 9th^51,59^. On December 11th, the FDA granted EUA for BNT162b2 in individuals >16 years old, while EMA and the European Commission approved the vaccine for individuals older than 16 years that reside in any of the 27 state members of the EU on December 21^st^ ^52,60^.

Several other countries have since given EUA for BNT162b2, Argentina, Chile, Ecuador, Costa Rica, Mexico, Panama, Kuwait, and Singapore among them. Switzerland, Bahrain, and Saudi Arabia have also authorized the vaccine, and on December 31st the WHO granted emergency validation to BNT162b2, thus allowing several countries to accelerate the processes of authorization, importation, and distribution of this candidate^61^.

On the same day, the safety and efficacy results of BNT162b2 Phase 3 clinical trial were published in the New England Journal of Medicine Apart from the confirmation of the 8 cases of COVID-19 among the participants assigned to receive BNT162b2 and 162 cases among those assigned to placebo an additional finding was revealed; in 10 reported cases of severe Covid-19 manifested after the participants had received the first dose, 9 occurred in the placebo group and only 1 in a BNT162b2 recipient, suggesting that BNT162b2 additionally protects from severe COVID-19.

### DNA vaccines

#### INO-4800 (Inovio/International Vaccine Institute)

Although Pennsylvania-based company Inovio has not yet entered officially Phase 3 trials their candidate is the most advanced SARS-CoV-2 DNA vaccine so far. Inovio Pharmaceuticals has developed several experimental DNA-based vaccines which are administered intradermally with the aid of a portable device called ‘Cellectra 2000’ that delivers a small electric pulse allowing for efficient cellular and nuclear uptake of the DNA molecules through an electroporation mechanism. Their candidate is a two-dose vaccine.

On June 30, a company announcement revealed interim data from a Phase 1 trial on 36 volunteers 18–50 years of age that receive two doses of either 1.0 mg or 2.0 mg of INO-4800 four weeks apart^62^. These results were published on December 23rd and according to the article, there were no serious adverse effects reported, and 34 out of 36 participants mounted strong humoral responses in both 1.0 mg and 2.0 mg groups^63^. Additionally, 78% of the participants in the 1.0 mg arm and 84% of the participants in the 2.0 mg arm generated neutralizing antibodies as assessed by a SARS-CoV-2/Australia/VIC01/2020 strain neutralization assay. Finally, 74% and 100% of the 1.0 mg and 2.0 mg immunized participants, respectively, developed strong Th1 and CD8+ T cell responses.

On September 28th, Inovio announced that the FDA had put the planned Phased 2/3 clinical trials of the vaccine candidate on a partial hold due to questions about the design and use of ‘Cellectra 2000’ ^64^. On November 16^th^, Inovio said that the F.D.A. had given them permission to move forward with their Phase 2/3 trial called INNOVATE^65^ (Clinical Trial Identifier: NCT04642638). The Phase 2 arm of this study will be carried out with 400 volunteers that will receive intradermally two-doses of either 1.0 or 2.0 mg of INO-4800 or a placebo with an interval of 4 weeks. The Phase 3 segment of the study will involve 6,178 volunteers that will receive doses determined by the safety and immunogenicity results obtained from the Phase 2 segment.

### Replication-defective viral vector vaccines

#### Ad5-nCoV (CanSino Biological/Beijing Institute of Biotechnology/Academy of Military Medical Sciences)

The Chinese company CanSino Biologics in collaboration with the Institute of Biology of China’s Academy of Military Medical Sciences developed a candidate using human adenovirus serotype 5 vector (Ad5) to deliver the information that codifies for SARS-CoV-2 full-length S protein into host cells. Ad5 is the main adenoviral serotype in humans, meaning that a significant percentage of individuals may have recent contact, and thus, pre-existing immunity against the viral vector that could hamper robust immune responses against the presented antigen as well.

In preclinical studies, immunization of BALB/c mice with one dose of Ad5-nCoV either intramuscularly or intranasally was found to induce strong humoral responses, including antigen-specific IgA production. Intranasal administration was shown to be more immunogenic and induce earlier peaking neutralizing antibodies than intramuscular vaccination as evaluated by a virus-specific microneutralization assay. Intranasally immunized mice where challenged with an HRB26M mouse-adapted SARS-CoV-2 virus shown no signs of viral replication in their lungs and turbinates or overt disease symptoms. Immunogenicity and viral challenge protection results were replicated in immunized ferrets that were exposed to wild-type SARS-CoV-2^66^.

Preliminary Phase 1 safety and immunogenicity data obtained from 108 participants between 18 and 60 years old who received low, medium, and high doses of Ad5-nCoV were published on May 22nd^67^. The two lower doses of 5 × 10^10^ and 1 × 10^11^ viral particles were found to have an acceptable safety and immunogenicity profile and were selected for a Phase 2 trial.

Results from the double blind randomised placebo-controlled Phase 2 trial were also published on July 20th^37^. Either of the two selected doses of Ad5-nCoV or the placebo were applied to a total of 508 eligible volunteers 18–83 years of age. Both dose groups elicited anti-RBD antibodies in more than 95% of the participants at day 28 post-immunization and neutralizing antibody titers against live SARS-CoV-2. Moreover, around 90% of vaccinated participants in both groups demonstrated activation of specific T cell responses as evidenced by interferon-γ ELISpot assay. Mild adverse reactions were reported by 72% and 74% of participants in the higher and lower dose groups, respectively. Severe adverse reactions were documented in less than 10% of participants of each group, and no serious adverse reactions were reported.

Following these results, the regimen of a single immunization of 5 × 10^10^ viral particles was selected to proceed in Phase 3 efficacy trials evaluating the protection from the incidence of severe COVID-19 with the enrollment of 40,000 volunteers in Saudi Arabia, Russia, and Pakistan (Clinical Trial Identifier: NCT04526990). Meanwhile, on June 25th, China’s Central Military Commission announced that Ad5-nCoV received approval for use in the military absent acquisition and analysis of Phase 3 trial results (Fig. 1).

#### AZD1222 (AstraZeneca/Oxford University)

The viral vectored vaccine of Oxford University and AstraZeneca represents another candidate. It was one of the first to begin clinical trials and the only one using a debilitated chimpanzee adenovirus (ChAdOx1) platform to circumvent the issue of pre-existing immunity against the vector, since very few -if any- humans would have a previous contact with a simian virus. The ChAdOx1 vector has been engineered to include the information that codifies for the wild-type SARS-CoV-2 Spike protein (Fig. 1).

Initial preclinical data on in animal models reveal a high immunogenic profile for AZD1222. More specifically, BALB/c and CD1 mice immunized with two doses of intramuscularly injected AZD1222 mounted strong humoral and cellular antigen-specific responses. Cytokine secretion profiling revealed that T-cell responses were significantly Th1-skewed. Furthermore, the authors went on to study the immunogenicity of AZD1222 in bigger animals. Therefore, they investigated if a ‘prime-only’ immunization regimen yielded similar results than a ‘prime-boost’ vaccination in pigs. The results obtained clearly showed that a ‘prime-boost’ immunization regimen enhanced neutralizing antibody titers in pigs as evaluated by a lentiviral-based SARS-CoV-2 pseudovirus neutralization assay, thus supporting the adoption of a prime-boost regimen for human clinical trials^68^. In line with these data, immunization of rhesus macaques with AZD1222 demonstrated that vaccinated animals produced both cellular and humoral responses that reduced their viral load in the lower respiratory tract when challenged with SARS-CoV-2 strain nCoV-WA1-2020. However, nasal swab samples demonstrated no significant reduction of viral loads in the vaccinated group, although the immunized animals exhibited no sign of clinically overt pathology^69^.

The first results of a Phase 1/2 single-blinded, randomised, multicentre control study of 1090 healthy adult volunteers aged 18–55 years was reported on July 20th^70^. A meningococcal protein compared with a meningococcal conjugate vaccine (MenACWY) served as control in this study. Participants received either one or two doses containing 5 × 10^10^ viral particles 4 weeks apart or the control meningococcal vaccine. The safety profile of the vaccine was characterized as acceptable while homologous vaccination provoked neutralizing responses against SARS-CoV-2 to all participants but had no additional effects on cellular adaptive responses.

Phase 3 efficacy and safety trials with the two-dose regimen (Clinical Trial Identifier: NCT04516746) is being carried out in more than 30,000 individuals in the U.S., India (Clinical Trial Identifier: CTRI/2020/08/027170), Brazil (Clinical Trial Identifier: ISRCTN89951424), Russia (Clinical Trial Identifier: NCT04540393) and South Africa. On September 6th, a serious adverse event (SAE) presented in a patient enrolled in Phase 3 studies temporarily halted the trials that resumed in most countries, including the UK, but not in the US. That was the second temporary pause for an AZD1222 study as in July the trial was halted for several days after a participant developed severe neurological symptoms. It was later concluded that these symptoms were due to a previously undiagnosed case of multiple sclerosis that was unrelated to the vaccine^71^. Primary endpoints of the Phase 3 study are focused on COVID-19 prevention and reactogenicity and tolerance profile of the candidate (Fig. 1).

On November 23th, Oxford University and AstraZeneca issued separate press releases presenting a Phase 3 interim results of their vaccine candidate^72,73^. These results were published on December 8th in Lancet in a safety and efficacy analysis of four trials carried out in Brazil, South Africa, and the UK thereby representing geographically and ethnically diverse populations^74^. The analysis included 131 COVID-19 confirmed cases detected in two different dosing regimens, including 11,636 participants recruited in the English and Brazilian arm of the study. The first regimen applied two full doses 4 weeks apart in 8895 adult participants and displayed a 62.1% efficacy. The second regimen, which was recognized to be the outcome of a logistics mistake, involved a half prime-dose followed by a full boost-dose with the same chronological separation between them and included 2741 individuals 18–55-year-old showing a 90% efficacy. It is hypothesized that this efficacy discrepancy might stem from the combination of the younger age group of the smaller cohort and the fact that a higher initial dose might promote the induction of antibodies against the viral vector, thereby hampering the intensity of the immune responses induced by the boost dose. The combined efficacy of the whole cohort of participants 14 days after the second dose was 70.4% without severe cases or hospitalizations being reported among vaccinated participants. Moreover, 3 weeks after the first dose was administered ten cases of hospitalization due to COVID-19 were reported, all in the control group. Preliminary data suggest that AZD1222 could reduce virus transmission since a reduction in asymptomatic infections was noted. On the efficacy arm it is reported that the safety profile of the vaccine candidate is generally good and that adverse reactions are less intense and frequent in older immunized participants that received lower doses and these events decline after the second dose.

AstraZeneca, with the support of Oxford University, submitted the complete interim Phase 3 safety and efficacy to several regulators including the UK, EMA, and Brazil for revision and emergency use approval of their candidate.

On November 27th, the MHRA issued a press release informing that the UK Department of Health and Social Care officially requested the review of the AZD1222 vaccine candidate and on December 30th was given an EUA for individuals >18 years old in the UK^75^ and Argentina^76^. On January 3rd, 2021, India granted an EUA to AZD1222 while on January 4, the first vaccinations with AZD1222 began at Oxford’s Churchill Hospital.

#### Gam-COVID-Vac/Sputnik V (Gamaleya Research Institute/Health Ministry of the Russian Federation/Acellena Contract Drug Research and Development)

Scientists of the Russian Research Institute Gamaleya developed the only heterologous prime-boost SARS-CoV-2 vaccine candidate thus far in order to circumvent the challenge of reduced immunogenicity due to antibodies raised against the viral vector after the first immunization. The adenoviral vector serotype used for the prime vaccination is different than the adenoviral serotype used as a booster. Hence, replication-defective Ad26 was selected to deliver the genetic information for Spike protein during the first vaccination and recombinant replication-defective Ad5 for the second.

The vaccine candidate, recently renamed Sputnik V, was tested in two small scale Phase 1/2 trials that involved 38 participants each. The results from the two studies were published on September 4th^77^, 3 weeks after President Putin had announced the authorization of Sputnik V for limited use. The Phase 1/2 trial report described a good safety profile with mild adverse effects in a portion of the vaccinated participants, such as asthenia, myalgia, arthralgia, fever, headache, and pain at the injection site. The immunogenic profile of the vaccine candidate was also good inducing strong humoral responses in all participants as well as CD4+ and CD8+ T cell activation.

Soon after the Phase 1/2 results were presented concerns were raised about the authenticity of data presented in several figures of the publication^77^. The additional fact that the Health Ministry of the Russian Federation has approved Sputnik V as the first vaccine for COVID-19 before Phase 3 safety and efficacy trials have caused controversy and concern in the scientific community.

Phase 3 trials were initially planned for just 2000 volunteers but later rescheduled to enroll 40,000 people at 45 different medical centres across Russia (Clinical Trial Identifier: NCT04530396) and the Republic of Belarus (Clinical Trial Identifier: NCT04564716). The primary endpoint is to demonstrate that Sputnik V prevents the vaccinated participants from COVID-19 development (Fig. 1).

On November 24th, the developers of Sputnik V announced the results from their second Phase 3 interim report that revealed a 91.4% efficacy of this vaccine candidate after analysing the data obtained from 18,794 people a week after receiving both doses of the heterologous immunization regimen^78^. This analysis is based on 39 confirmed COVID-19 cases among the participants, 8 of whom were reported to belong in the vaccinated group and 31 in the placebo group. According to the press release, no life-threatening (Grade 4) adverse events were detected, whilst the most common severe (Grade 3) events reported were pain at the injection site and flu-like symptoms such as fever, fatigue, and headache.

On December 14th, the Gamaleya National Center and one of its sponsors, the Russian Direct Investment Fund (RDIF), announced the results of their final interim analysis after reaching the checkpoint of 78 confirmed COVID-19 cases^78^. The efficacy of Sputnik V three weeks after administering the first dose was reported to be 91.4% based on the analysis of data obtained from 22,714 participants (17,032 received the vaccine and 5682). In the immunized arm 16 cases of COVID-19 were reported versus 62 cases in the placebo arm. Moreover, the announcement reported 20 severe cases of COVID-19 in the placebo group and no severe disease cases in the vaccinated group, claiming that the vaccine demonstrated 100% efficacy against severe COVID-19.

Apart from the early use approval of Sputnik V granted in Russia, EUAs for this vaccine have also been issued from Belarus and Argentina.

#### JNJ-78436735/Ad26.COV2.S (Janssen and Beth Israel Deaconess Medical Center)

Janssen Pharmaceuticals is the vaccine development branch of Johnson & Johnson pharmaceutic. Their candidate is a replicating-defective adenovirus 26 based vector expressing the stabilized pre-fusion S protein of SARS-CoV-2, a method developed a decade ago by researchers of the Beth Israel Deaconess Medical Center in Boston. Their main difference from the CanSino vaccine candidate is the adenovirus serotype. As opposed to the ubiquitous Ad5 serotype, very few people have been exposed to the rare Ad26 serotype, therefore, pre-existing immunity against the vector reducing this candidate’s immunogenicity is not expected to be a major concern. The second advantage of this candidate is that the dosing schedule involves a single immunization.

Preclinical studies were initially carried out in Syrian golden hamsters. When intranasally challenged with high viral loads of SARS-CoV-2 a subset of these animals develops severe pathology and symptoms mirroring severe COVID-19 in humans. Intramuscular immunization of hamsters with a single injection of the Ad26-vectored vaccine candidate resulted in the production of high titers of ant-RBD and virus neutralizing antibodies as evaluated by a SARS-CoV-2 pseudovirus assay. In this assay SARS-CoV-2 pseudoviruses carry a luciferase reporter gene and the production of luciferase by pseudovirus-exposed Vero E6 cells is assessed before and after pre-incubation with animal sera. Moreover, immunization with the Ad26-vectored vaccine candidate prevented severe disease development and mortality in hamsters challenged with the USA-WA1/2020 strain of SARS-CoV-2^79^. Further studies on rhesus macaques demonstrated that after a single dose of the vaccine animals mounted strong antibody and T-cell mediated responses. Antibodies produced demonstrated viral neutralization potential when sera were examined in pseudovirus neutralizing assays, whilst CD8+ and CD4+ T-cell responses showed a Th1-skewed phenotype as assessed by cytokine profiling. Finally, animals receiving the Ad26-S.PP version (stabilized pre-fusion form of Spike protein induced by two proline mutations) of the vaccine candidate did not develop disease pathology or detectable viral load in bronchoalveolar lavage samples following SARS-CoV-2 exposure^80^, suggesting this candidate warrants clinical study assessment.

Based on these findings, Janssen launched a multi-centre randomized, double-blinded, placebo-controlled Phase 1/2 trial in July whose results were recently reported^81^.

As with its viral-vectored counterparts. JNJ-78436735 was administered at either dose levels of 0.5 × 10^11^ or 1 × 10^11^ viral particles per vaccination in participants of two different age groups: the first group was comprised of 402 healthy adults ranging from 18 to 55 years old and the second group was comprised of 394 healthy elderly 65 years or older.

The reactogenicity of the vaccine was mild, mainly causing injection site pain, fever, headache, and myalgia. Specific antibodies against S protein were detected in 92% of participants of the younger group that received either dose and reached 100% seroconversion rate in the older group. CD4+ T cell responses were observed in more than 80% of the members of either group and robust CD8+ T cell responses were also documented.

These results prompted a Phase 3 trial recruiting up to 60,000 participants that will receive the dose level of 0.5 × 10^11^ viral particles (Clinical Trial Identifier: NCT04505722). The trial’s primary to analyzed outcomes were occurrence of moderate to severe COVID-19.

On October 12th, the Phase 3 clinical trials of Janssen paused after a study participant manifested a SAE. No data have been released as to what was the nature of the illness or if the participant had received the vaccine candidate or the placebo, but on October 23rd, Jannsen announced that their the Phase 3 trial of their candidate was about to resume after the recommendation of the independent Data Safety and Monitoring Board (DSMB) supervising the study^82^.

On November 15th, Janssen informed that they will initiate a second Phase 3 randomized, double-blind, placebo-controlled clinical trial studying the safety and efficacy of a two-dose regimen of their candidate (Clinical Trial Identifier: NCT04614948). The study will involve 30,000 adult participants from Belgium, Colombia, France, Germany, the Philippines, South Africa, Spain, the United Kingdom and the United States that will receive either two doses of the Ad26.COV2.S vaccine candidate or a placebo with a 57-day interval^82^.

### Inactivated pathogen vaccines

#### CoronaVac (Sinovac Research and Development Co.)

Initially launched under the name PiCoVacc, CoronaVac, it is a purified, inactivated virus alum-adjuvanted candidate vaccine. The candidate was produced by β-propiolactone-activation of the CN2 strain of SARS-CoV-2 isolated from the bronchoalveolar lavage of a hospitalized patient^83^, this strain is closely related to the 2019-nCoV-BetaCoV Wuhan/WIV04/2019 strain. Inactivated vaccines present some technical challenges as a disadvantage. The inactivation process can sometimes damage the antigens leading to suboptimal immunogenicity. Also, inactivated vaccines commonly need several boost doses to produce strong immune responses and do not traditionally activate cellular responses. More importantly, in order to enhance their capacity to provoke immunity, these vaccines require the addition of adjuvants. Indeed, CoronaVac is an alum-adjuvanted candidate vaccine.

Gao et al. described that their candidate was highly immunogenic in BALB/c mice and Wistar rats inducing high levels of anti-S and anti-RBD antibodies that peaked 6 weeks post-immunization but, surprisingly, induced lower anti-Nucleocapsid protein antibody titers. Among the elicited antibodies, SARS-CoV-2 specific neutralizing antibodies were detected as assessed by microneutralization assays that measure the immunized animal sera capacity to inhibit the SARS-COV-2 cytopathic effects on Vero cells.

Moreover, immunization of rhesus macaques (Macaca mulatta) with CoronaVac and their subsequent exposure to SARS-CoV-2 three weeks post-immunization revealed that the vaccine candidate induced partial or complete protection against the viral challenge as assessed by viral RNA titers in pharynx, crissum, and lung histopathological changes^83^.

A Phase 2 randomized, double-blind, placebo-controlled dose-escalation safety and immunogenicity trial was conducted enrolling 600 healthy volunteers between 18 and 59 years old who received two different dosages of the vaccine (3 or 6 µg/0.5 ml) or placebo^84^. CoronaVac was well tolerated at both dosages and most of the adverse reactions were mild. Pain at the injection site was the most common of reported symptoms. Both CoronaVac dosages induced a seroconversion on more than 90% of the individuals immunized, however, no T cell responses were reported.

CoronaVac is currently in Phase 3 clinical trials in a two-dose injection regimen with a 14-days interval. These trials will enrol 8870 participants from Brazil (Clinical Trial Identifier: NCT04456595), volunteers from Indonesia (Registration Number: INA-WXFM0YX), and Turkey^85^ evaluating the efficacy of the vaccine in preventing from COVID-19 and the frequency of adverse effects.

CoronaVac reportedly received an emergency approval for limited use in July in China, whilst Sinovac is scaling up production of their candidate vaccine with an aim to supply Indonesia with 40 million doses by March 2021 and begin worldwide distribution in early 2021 (Fig. 1).

#### Unknown name (Wuhan Institute of Biological Products/China National Biotech Group-Sinopharm)

The Wuhan Institute of Biological Products teamed up with state-owned Sinopharm pharmaceutical to develop a purified, inactivated virus vaccine that was submitted for Phase 1 and Phase 2 clinical trials. This candidate was developed by initially isolating the WIV04 strain of SARS-CoV-2 from a patient in the Jinyintan Hospital, Wuhan. The virus was then propagated in a Vero cell line and inactivated with β-propiolactone. Finally, the vaccine was subjected to an alum-adjuvant adsorption procedure.

The Phase 1 and Phase 2 trials interim report on healthy adults between 18 and 59 years old was published on August 13^86^. In the Phase 1 dosage and safety trial, 96 participants received one of three different dosages (2.5, 5, and 10 μg/dose) or an alum adjuvant-only control in a three-injection regimen. In the Phase 2 safety and immunogenicity trial, 224 adults were randomly assigned to receive twice a 5 μg/dose immunization with a 2- or 3-week interval between each dose or receive the adjuvant-only control. The results showed that the vaccine candidate had good safety profile with only mild adverse effects documented (mainly injection site pain and fever), produced antibodies in volunteers, some of whom experienced fever and other side effects. Both groups that received the candidate vaccine produced high titers of neutralizing antibodies against SARS-CoV-2, although the titers were significantly higher in the group that received the two injection 3 weeks apart.

Phase 3 clinical trials are currently being carried out in the United Arab Emirates (Registration Number: ChiCTR2000034780), Peru, Morocco (Registration Number: ChiCTR2000039000) and Bahrain (Fig. 1)^87^.

#### BBIBP-CorV (Beijing Institute of Biotechnology/ China National Biotech Group-Sinopharm)

The second inactivated virus vaccine candidate developed by Sinopharm is the result of their collaboration with the Beijing Institute of Biological Products (Fig. 1). 2.3.3. BBIBP-CorV was developed by β-propiolactone-mediated inactivation of the 19nCoV-CDC-Tan-HB02 strain SARS-CoV-2 that was replicated in Vero cells^88^ and adjuvanted with aluminium hydroxide. Aluminium hydroxide activates the NLRP3 receptor subunit of the inflammasome and promotes the secretion of high-levels of inflammasome-derived IL-1β and IL-18, thus activating proinflammatory mechanisms of the immune system^89^.

Preclinical studies on animal models showed that the aluminium hydroxide-adjuvanted vaccine candidate induced the production of high levels of neutralizing antibodies titers against SARS-CoV-2 as calculated by microtitration experiments. In these experiments, animal sera were serially diluted and preincubated with a stable concentration of SARS-CoV-2 in 96-well culture plates. Afterward, Vero cells were added to the preincubated wells and the highest dilution of serum that was found to can protect 50% of cells from SARS-CoV-2 infection is the antibody potency of the serum. Additionally, vaccination with BBIBP-CorV was shown to confer protection against SARS-CoV-2 intratracheal challenge in rhesus macaques 7 days post-vaccination as evidenced by throat and anal swabs viral loads, lung tissue viral load calculation and pathological examination results^90^. Results from a Phase 1/2 randomised, double-blind, placebo-controlled study were published on October 15th^88^.

In dose escalation and safety Phase 1 study, 192 participants received either 2 μg, 4 μg or 8 μg of the vaccine or the placebo. Two age groups were employed, namely 18–59 years and ≥60 years. The most common systematic adverse effect reported was fever in less than 10% of the candidates. The safety profile of the vaccine was quite good as all adverse reactions documented were mild or moderate and no serious events were reported. The immunogenicity of the candidate was higher in the younger age group (18–59 years) and neutralising antibody titres presented a dose dependent induction.

In Phase 2 trials, 448 volunteers were recruited and received either one dosage of 8 μg or two dosages of 4 μg of vaccine 2, 3 or 4 weeks apart. Again, adverse reactions reported were mild or moderate and the most frequent systematic reaction was fever in less than 4% of the members of each dosage group. Neutralising antibody titres were significantly higher in the groups that received a prime-boost immunization with 4 μg/dose and highest when the two immunizations were separated by a 3-week distance. As expected, no cellular immune responses were reported.

At this moment BBIBP-CorV is in Phase 3 clinical trials in Argentina (Clinical Trial Identifier: NCT04560881), Bahrain, Jordan, Egypt, and U.A.E. (Clinical Trial Identifier: NCT04510207), (Registration Number: ChiCTR2000034780) evaluating the incidence of COVID-19 in individuals that have received two doses of the vaccine.

Both Sinopharm’s vaccine candidates are reportedly scheduled to be ready for market by the end of the year^91^. On September 14th, the U.A.E. gave emergency approval for Sinopharms’ vaccines to be administered to health care workers, before obtaining large scale data of their safety and efficacy^92^. On December 9th, the U.A.E. granted full approval to BBIBP-CorV, followed by Bahrain on December 13th^93,94^. On December 30th Sinopharm announced that their candidate had a 79.34% efficacy and received approval in China the following day.^95^ On the other hand, Egypt issued an emergency authorization on January 3rd, 2021^96^.

#### Covaxin/BBV152 (Bharat Biotech/ Indian Council of Medical Research/ National Institute of Virology)

India-based Bharat Biotech and the Indian Council of Medical Research developed a purified inactivated whole virion vaccine candidate named Covaxin. The vaccine was developed by β-propiolactone inactivation of an Indian strain of the novel coronavirus isolated by the Indian National Institute of Virology and propagated in Vero CCL-81 cells. An alum-adjuvanted version of the vaccine candidate was found to significantly reduce or nullify viral loads and bronchoalveolar affection in rhesus macaques challenged with SARS-CoV-2 14 days after receiving the second dose of the vaccine candidate, as attested by viral load measurement in bronchoalveolar lavage fluid, nasal swab, throat swab, and lung tissues at 7 days post-infection in the immunized animals. Furthermore, no signs of pneumonia were detected in histopathological sections of the vaccinated and subsequently virus-challenged animals^97^. Moreover, the authors found that a combination of alum and imidazoquinoline used as an adjuvant significantly potentiated the immunogenicity of the vaccine. Alum mainly acts as an inducer of NALP3-inflammasome^98^, whilst imidazoquinoline is a Toll-like receptor 7 and 8 agonist.

A preprint of a Phase 1 clinical trial of BBV152 was released on December 15th^99^. The authors report results obtained from 375 participants that received three different formulations of BBV152 (*n* = 100 per each formulation) or the Algel (aluminium-based) adjuvant (*n* = 75). The three different formulations included 3 or 6 μg of whole-virion inactivated SARS-CoV-2 adsorbed to alum (Algel-imidazoquinoline) or 6 μg of whole-virion inactivated SARS-CoV-2 adsorbed to Algel alone. Participants received two intramuscular doses 2 weeks apart and the safety and immunogenicity of each formulation was assessed. Adverse effects were found to be mild or moderate with an incidence rate between 10 and 20% and pain at the injection site being the most common reported event. In both algel-imidazoquinoline groups induction of high titers of anti-SARS-CoV-2 antibodies, CD4+ and CD8+ T cells was reported and were significantly higher than the algel-only group. T cell responses appeared to be Th1-biased whilst seroconversion rates after the second dose were 87.9% and 91.9% for the 3 and 6 μg groups, respectively. Additionally, neutralizing antibody titers against SARS-CoV-2 and seroconversion rates were assessed both by a microneutralization assay using SARS-CoV-2 and a plaque-reduction neutralization test using three different strains for viral challenge. Neutralizing antibody titers were significantly higher in the two alum-imidazoquinoline groups than the algel-only vaccine group, while neutralizing seroconversion rates were 93.4% and 86.4% in the 3 and 6 μg adjuvanted with alum-imidazoquinoline groups, respectively versus 86.6% in the 6 μg algel-only group.

A Phase 3 randomized, double-blind, clinical study to evaluate efficacy, safety and immunogenicity of this candidate began on October 23rd (Registration Number: CTRI/2020/11/028976) in India. A total number of 25,800 adult participants will receive 3 μg of the adjuvanted form of the candidate or adjuvanted phosphate buffered saline with as a placebo, in a prime-boost regimen of two intramuscular immunizations separated by 4 weeks. The vaccine candidate contains 6 µg of inactivated virus per vaccination. Initial results from the Phase 3 clinical trial are expected in the first trimester of 2021.

On January 3^rd^, 2021, India granted an EUA to BBV152 although participants are still recruited for the candidates’ Phase 3 safety and efficacy trials.

### Protein subunit vaccines

#### NVX-CoV2373 (Novavax)

Similar to inactivated pathogen vaccines, protein subunit candidates usually exhibit an extremely favorable safety profile but require multiple boost doses and elicit low grade cellular responses.

Maryland-based Novavax has developed a prefusion full-length recombinant SARS-CoV-2 S glycoprotein nanoparticle expressed in a baculovirus-Sf9 system and is administered with an adjuvant named Matrix M1. Saponin based Matrix M1 adjuvant is used precisely to tackle the absence of cell mediated immune responses that characterize protein subunit vaccines^100^.

Matrix M-adjuvanted NVX-CoV2373 was first investigated in animal models, such as rats and baboons to assess immunogenicity. Indeed, addition of the adjuvant was found to significantly enhance antibody production in immunized BALB/c mice and induce strong T-cell responses that exhibited a Th1-skewed phenotype. Administration of a two-dose regimen of Matrix M-adjuvanted NVX-CoV2373 elicited high titer antibodies that were shown to efficiently neutralize in vitro the cytopathic effects of SARS-CoV-2 on Vero E6 cells and also to prevent the infection of mice transfected to express the human ACE2 receptor with SARS-CoV-2. Moreover, these results were replicated in olive baboons receiving intramuscularly two doses of Matrix M-adjuvanted NVX-CoV2373 with an interval of 3 weeks^101^.

Additional studies on cynomolgus macaques (*Macaca fascicularis*) immunized with Matrix M-adjuvanted NVX-CoV2373 and later exposed to intranasal and intratracheal challenge with the 2019-nCoV/USA-WA1/2020 strain of SARS-CoV-2 were found to produce high levels of anti-S neutralizing antibodies that protected them both against upper and lower respiratory tract infection and pulmonary disease. Neutralizing antibody levels were calculated with a cytopathic effect assay of SARS-CoV-2 in Vero E6 cells^102^.

After these preclinical promising results in animal models, Novavax launched a Phase 1/2 trial whose results were published in the New England Journal of Medicine on September 2nd^103^. A total 131 healthy adults were randomly assigned to receive two administrations of the vaccine with or without the adjuvant or a placebo. The adverse effects produced were null or mild and of short duration. The addition of adjuvant enhanced the immune responses elicited by the vaccine candidate and resulted in cellular responses that exhibited a Th1-skewed phenotype. Anti-S IgG and neutralizing antibodies induced by vaccination exceeded those detected in convalescent sera from COVID-19 patients.

On September 23rd, Novavax launched a Phase 3 trial that aims to enrol up to 9000 volunteers in the United Kingdom (Registration Number: 2020-004123-16) and is planning to expand it in the US, India, and other countries (Fig. 1). During the same month, Novavax established collaboration with the Serum Institute of India that will enable the production of up to 2 billion doses a year. On December 28th, Novavax initiated a Phase 3 trial in the US aiming to recruit 30.000 participants half of whom will receive 5 μg of prefusion full-length recombinant SARS-CoV-2 S glycoprotein nanoparticle adjuvanted with 50 μg of Matrix M1 (Clinical Trial Identifier: NCT04611802).

From the list of the ten leading vaccine candidates five (mRNA-1273, mRNA-BNT162b2, AZD1222, JNJ-78436735, NVX-CoV2373) are part of Operation Warp Speed, an initiative that has set the goal to deliver 300 million doses of safe and efficient vaccines by mid-2021 in the U.S.

#### ZF2001 (Anhui Zhifei Longcom Biopharmaceutical/Chinese Academy of Medical Sciences)

The latest subunit vaccine candidate to enter Phase 3 clinical studies is the adjuvanted RBD-dimeric antigen designed by Anhui Zhifei Longcom Biopharmaceutical and the Institute of Microbiology of the Chinese Academy of Medical Sciences. Phase 3 clinical study was launched on December^104^ and will be initially carried out in China and Uzbekistan while Indonesia, Pakistan and Ecuador will follow as study sites (Clinical Trial Identifier: NCT04646590 and Registration Number: ChiCTR2000040153). The design of the study involves recruitment of 22,000 volunteers from China and 7000 subjects outside China for a total of 29,000 volunteers. There are still no published results on this candidate, however data from its Phase 2 placebo-controlled clinical trial (Clinical Trial Identifier: NCT04466085) conducted on a total of 900 participants ranging from 18 to 59 years old suggest that a 2 or 3 dose regimen is evaluated. Each immunization will be separated by the next by 4 weeks.

#### Unknown name (Sanofi Pasteur/GlaxoSmithKline)

This candidate is designed in a similar fashion as the FluBlok quadrivalent vaccine produced by Sanofi. Sanofi uses a baculovirus expression system to transfer the genetic information of the immunogen in lepidopteran insect cells that subsequently express high levels of the codified antigen^105^, in this case, the S protein of SARS-CoV-2. GSK is providing their AS03 (Adjuvant System 3) squalene-based adjuvant that has been successfully used in various vaccines developed by GSK such as the pandemic influenza A (H1N1) vaccine called Pandemrix^106^. On September 3rd, Sanofi announced that they enter combined Phase 1/2 clinical trials with their vaccine candidate^107^. This trial was conducted with the participation of 440 participants across 11 investigational sites in the United States (Registry Number: NCT04537208). Participants were divided into three different groups and received one or two doses of the vaccine, or a placebo control, respectively. However, on December 11th Sanofi and GSK announced that their candidate failed to elicit strong immune responses in participants older than 50 years and only showed efficacy in adults aged 18–49 years. Thus, the two companies plan to optimize the antigen concentration administered by their candidate to improve its immunogenicity and initiate a Phase 2b with a new formulation that will be compared against an already authorized SARS-CoV-2 vaccine^108^.

### Virus like particle vaccine

#### CoVLP (Medicago)

Virus like particle vaccines aim to combine the efficacy of attenuated pathogen vaccines with the excellent safety profile usually found in subunit vaccines. The VLP displays multiple copies of the target antigen on its surface and has a size that favors recognition and subsequent uptake from antigen-presenting cells, therefore promoting its efficient phagocytosis, processing, and presentation by dendritic cells, and inducing strong adaptive responses.

The only advanced candidate against SARS-CoV-2 that employs this strategy is the vaccine designed by the Quebec-based company Medicago. Medicago’s approach is unique as it uses the virus-transfected plant *Nicotiana benthamiana* to express the prefusion trimeric subunit form of the SARS-CoV-2 S-protein and assemble it on the surface of VLPs which are harvested and used for immunization.

A Phase 1 clinical study was carried out after enrolling 180 participants, 18–55 years old, that were subjected to a two-vaccination regimen of either of three doses (3.75 µg, 7.5 µg, and 15 µg) per dose. Moreover, each of the doses was supplemented with either of CpG 1018 or AS03 adjuvants or was applied without an adjuvant. CpG 1018 is a ligand of Toll-like receptor 9 developed by Dynavax that induces robust cellular and humoral responses^109^, while the squalene-based AS03 adjuvant is developed and patented by GSK and has been used in several of the company’s products^110^. Results of this studies were published online as a pre-print on November 6th^111^ and indicate that administration of CoVLP was well tolerated as it created only mild to moderate transient adverse events. Moreover, there was no dose-dependent effect on neutralizing antibodies’ induction and addition of both adjuvants induced strong humoral and cellular responses. However, when administered together with AS03, even the lowest, 3.75 μg dose of the vaccine candidate was able to elicit strong T-cell responses and neutralizing antibody levels that were approximately 10-times higher than those produced on average in Covid-19 convalescent individuals.

Based on these results, a randomized, observer-blind, placebo-controlled Phase 2/3 began recruitment on November 19th (Clinical Trial Identifier: NCT04636697). The study will include 30,612 adult volunteers that will receive 2 intramuscular doses of 3.75 µg of CoVLP vaccine adjuvanted with 0.5 mL of AS03 adjuvant or a placebo with a difference of 3 weeks.

## Discussion

Several laboratories and pharma companies worldwide are developing an effective vaccine against COVID-19. Several vaccine candidates have been developed using different platforms and it seems we are close to witnessing the first emergency use approval for a SARS-CoV-2 vaccine. In fact, on December 2nd, the Pfizer/BioNtech vaccine become the first vaccine to receive approval for emergency use in the UK. Since then, the Moderna and Oxford/AstraZeneca vaccines have also received EUAs from several drug regulating agencies and vaccinations have began in several countries. Earlier, in the third trimester of 2020 other leading candidates had also been granted approvals for limited use in China, Russia, and U.A.E.

The vast majority of SARS-CoV-2 vaccines under development require a prime-boost regimen. Massive vaccination campaigns would therefore require billions of doses to satisfy global demand. For the first time in vaccine development history pharma companies are scaling up at risk production, without knowing if their candidate will receive authorization, but in theory will still require years to produce these numbers. This means that since the first candidates were authorized for wide use governments had to chart prioritization strategies. High vulnerability groups such as health workers and indispensable professionals are the first to receive a vaccine, followed by age groups older than 65 years^112,113^. On the other hand, more strategic alliances are constantly forming between pharmaceuticals and institutions to increase SARS-CoV-2 vaccine production sites worldwide and maximize the production of vaccines on large scale to meet global demand.

Cold chain issues for different platforms can also be a decisive factor for their widespread use. Nucleic acid and -sometimes- viral vector platforms that require long-term storage at −70 °C from fabrication to administration can raise severe problems for the distribution of the respective vaccines and limit their use in rural areas.

Another caveat is that we have not yet defined which the correlates of protection against COVID-19 or SARS-CoV-2 infection are. It is important to pinpoint the exact antibody titers that confer protection or the details of T cell responses that result in asymptomatic or mild disease. Knowing the correlates of protection will provide us with specific measurable aspects of immune response needed to thwart severe disease or even prevent infection. Further research is also needed to explore the durability of the immunity induced by each vaccine. We already know that infection by human coronaviruses produces humoral immunological memory that ranges from months to a couple of years, but long-term data on SARS-CoV-2 immunity are still lacking.

An additional consideration is the absence of children and pregnant women -and other vulnerable groups- from clinical trials conducted so far. It is quite probable that these groups will have to wait for additional small-scale clinical trials after the first generation of vaccines has been approved for other adult groups.

A possible problem might be that the first generation of SARS-CoV-2 vaccines will probably not confer sterilizing immunity against SARS-CoV-2 as current Phase 3 trials are evaluating candidate efficacy for disease prevention rather than infection prevention.

All the leading vaccine candidates are administered via intramuscular injection. However, results emerging from several recent studies highlighting the importance of mucosal immune responses against SARS-CoV-2 infection^114,115^. These suggest that intranasal administration as a more attractive strategy to marshal early protective immune responses in the upper respiratory tract mucosa before SARS-CoV-2 gains a foothold in the lower respiratory tract. Several vaccine candidates are exploring this potential. Accordingly, Maryland-based company Altimmune is recruiting volunteers for a Phase 2 clinical trial with their intranasally administered vaccine candidate called AdCOVID (Clinical Trial Identifier: NCT04442230), whereas China will begin Phase 1 clinical trials on an intranasally administered vaccine candidate for COVID-19 as announced on early September^116^. AdCOVID is an adenovirus type 5 (Ad5)-vectored vaccine encoding the RBD of the SARS-CoV-2 S protein. In a recent pre-print, AdCOVID intranasal administration was found to confer protective immunity in murine models eliciting robust cellular and humoral immune responses against RBD and resulting in the production of mucosal IgA^117^. Additionally, San Francisco-based Vaxart has also designed an Ad5-based oral SARS-CoV-2 candidate given in a form of a tablet. When they tested a candidate that encodes full-length S protein of SARS-CoV-2 in mice they detected induction of higher titer SARS-CoV-2 specific antibodies both in the bloodstream and in the lungs, along with the production of antigen-specific CD4+ and CD8+ T cells^118^. On September 21st, Vaxart’s candidate named VXA-CoV2-1 entered Phase 1 studies enrolling 48 healthy adult volunteers aged 18–54 years old (Registration Number: NCT04563702). Merck and IAVI are also developing a recombinant VSV viral-vectored SARS-CoV-2 vaccine in the form of a tablet. This approach is similar to the ERVEBO vaccine against Ebola designed by Merck. On October 30th, V590—as is the current candidate’s name—entered Phase 1 trials and will be administered to 252 volunteers aged 18–54 years old (Registration Number: NCT04569786). Finally, Canada-based company Symvivo announced on November 2nd, that they began a Phase 1 clinical trial using a DNA vaccine platform, whereby the DNA is inserted and replicated in probiotic bifidobacteria which then are administered orally in a liquid form, delivering the DNA codifying for the S protein of SARS-CoV-2 in intestinal epithelial cells, which then express and present the viral protein. The bacTRL-Spike vaccine candidate will be delivered in 12 participants in different concentrations (Registration Number: NCT04334980) that will be studied for safety and immunogenicity parameters.

Additional clinical trials are conducted with existing vaccines that do not specifically target SARS-CoV-2, but instead aim to activate trained immunity responses that could partially protect against SARS-CoV-2 infection or disease severity.

Since the first SARS-CoV-2 vaccines received emergency use approvals, a race against the clock has begun to provide an enormous number of doses and immunize vulnerable populations globally in a prioritized fashion. However, this titanic effort might be curtailed by vaccine hesitancy. Indeed, surveys of vaccination intention have shown that in the US only 40-50% of the general population plan to be vaccinated once a SARS-CoV-2 vaccine will be made available^119^ and this problem needs to be addressed promptly.

In spite of that, the only strategy to achieve herd immunity for COVID-19 and be able to return to pre-pandemic normality is a safe and efficient vaccine that would not only be able to prevent severe COVID-19 symptomatology but minimize viral load or even prevent infection and generate immune memory responses for a minimum of 1 year.

## Conclusions

The world is in the midst of a COVID-19 pandemic and the entire vaccinology scientific community is racing to find a vaccine against SARS-CoV-2 that is safe and effective. There are currently more than 230 vaccine candidates under development, with a number of these already receiving EUAs within less than a year since the first report of a SARS-CoV-2 infection.

Ethics committees are revising their authorization protocols, and pharmaceutical companies have formed strategic alliances with vaccine developing institution in order to ramp up production of vaccine candidates at risk. More than 150 countries have entered the COVAX initiative and other alliances that will aim to ensure an equitable distribution of an approved vaccine.

Several governments have made up-front payments to secure a number of doses of the vaccines under development that will help to return to a pre-COVID-19 normality.

However, several aspects of anti-SARS-CoV-2 immunity are still unknown and more specific conclusions about the correlates of protection against SARS-CoV-2 infection are expected to be drawn together with the initial results of Phase 3 trials. The vast majority of the vaccine candidates aim to elicit neutralizing antibodies against the S protein of the virus, thereby inhibiting the viral particle recognition and uptake mediated by human ACE2 receptor binding. Indeed, the majority of published clinical study results compare the neutralizing antibodies elicited with by immunization with the vaccine candidates against the neutralizing antibody levels averagely produced in convalescent COVID-19 individuals. In this regard, most preliminary results are quite promising as different candidates were found to induce higher neutralizing antibody titers than natural infection. An increasing body of evidence suggests that T cell mediated response is an arm of coronavirus immunity. Accordingly, vaccine platforms that also activate this arm of adaptive immunity, such as viral vectored or nucleic acid vaccines, are gaining favor among experts.

Developing multiple vaccine candidates that employ different vaccine delivery systems will probably prove to be crucial in the fight to end the COVID-19 pandemic. On one hand, several vaccination options, if approved, will enable us to produce the necessary doses for massive vaccination in a shorter timeframe. On the other hand, it is quite possible that different vaccine platforms will exhibit different grades of protection against specific population groups with altered immune responses such as children, pregnant women, immunocompromised populations due to comorbidities, and immunosenescent age groups ≥65 years.

Meanwhile, several clinical trials are exploring whether already approved vaccines can confer a certain grade of protection against COVID-19. The Bacillus Calmette–Guérin (BCG) vaccine and the MMR (measles, mumps, and rubella) vaccine are known to elicit strong immune responses with the activation of both specific and non-specific T cell populations. This bystander activation of heterogeneous T cell populations along with trained innate immunity mechanisms have been shown before to protect against viruses of the respiratory tract.

Finally, several critical aspects of SARS-CoV-2 immunity will be elucidated as a result of massive vaccination campaigns. The durability of the immunity induced by the different vaccine strategies as well as the fine details of the immune responses elicited will emerge as bigger populations get vaccinated, including individuals with suboptimal immunity.

## Data Availability

All data generated or analyzed during this study are included in this published article.
